# 446. Post-Acute Sequelae of COVID-19 in a Rural Wisconsin Community Prospective Cohort

**DOI:** 10.1093/ofid/ofad500.516

**Published:** 2023-11-27

**Authors:** Maria Sundaram, Oluwakemi Alonge, Joshua Petrie, Leora R Feldstein, Melissa A Rolfes, Yoshihiro Kawaoka, Gabriele Neumann, Edward Belongia, Huong McLean

**Affiliations:** Marshfield Clinic Research Institute, Marshfield, Wisconsin; Marshfield Clinic Research Institute, Marshfield, Wisconsin; Marshfield Clinic Research Institute, Marshfield, Wisconsin; Centers for Disease Control and Prevention, Atlanta, GA; Centers for Disease Control and Prevention, Atlanta, GA; University of Wisconsin, Madison, WI; University of Wisconsin, Madison, WI; Marshfield Clinic Research Institute, Marshfield, Wisconsin; Marshfield Clinic Research Institute, Marshfield, Wisconsin

## Abstract

**Background:**

Some individuals experience post-acute sequelae of SARS-CoV-2 (PASC), a variety of persistent symptoms related to different organ systems after a SARS-CoV-2 infection. There are limited data on the prevalence of PASC after SARS-CoV-2 Omicron variant infection. We assessed the relative risk of persistent symptoms consistent with PASC, during a period of SARS-CoV-2 Omicron circulation.

**Methods:**

A longitudinal cohort in rural Wisconsin was followed from November 2020 – July 2022 to assess incidence of SARS-CoV-2 infection. In March – April 2023, participants completed a survey about ongoing symptoms lasting ≥ 4 consecutive weeks and beginning on or after January 2022 (Omicron period). Poisson regression models were used to assess the relative risk of persistent symptoms overall and by symptom category among those with RT-PCR-confirmed or self-reported SARS-CoV-2 infection since January 1, 2022 (Omicron infection) vs. no history of SARS-CoV-2 infection (by self-report or RT-PCR detection), controlling for participant age group (17-49 vs. ≥50 years), having ≥2 COVID-19 vaccine doses before 2022 (yes/no) and chronic health conditions (yes/no).

**Results:**

Of 573 respondents aged ≥17 years (66% response rate), 63% were female, 60% were ≥50 years old, and 51% had a chronic health condition. The presence of ≥2 persistent symptoms was 27% among Omicron-infected individuals and 24% among never-infected individuals (28% for those with SARS-CoV-2 infection prior to 2022). The risk of having ≥2 persistent symptoms was similar for Omicron-infected vs. never-infected individuals (adjusted relative risk [aRR]: 1.1; 95% CI: 0.9, 1.4). In models investigating symptom categories, aRRs for cardiac, pulmonary, neurologic, and menstrual symptoms were elevated (but not statistically significant) for Omicron-infected vs. never-infected individuals (**Figure 1**).

**Figure 1.**
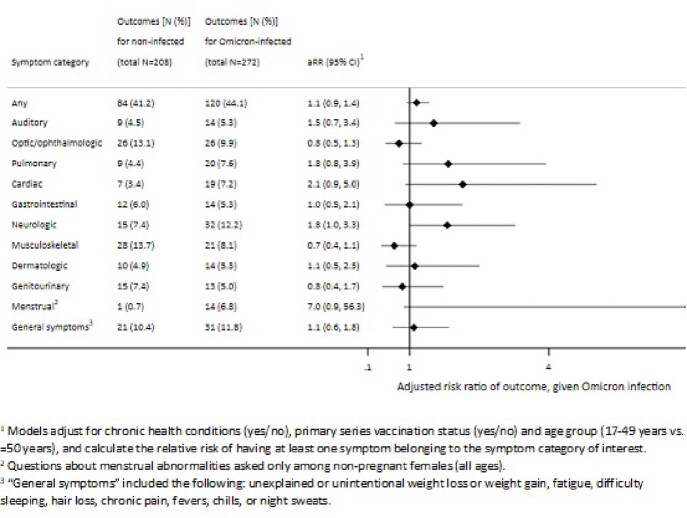
Relative risk of PASC-related symptoms by category among individuals with Omicron infection, compared to individuals with no history of SARS-CoV-2 infection.

**Conclusion:**

Preliminary data from this cohort show presence of persistent symptoms with and without a history of SARS-CoV-2 infection. We observed elevated though non-significant aRRs for cardiac, pulmonary, and neurologic symptoms for Omicron-infected individuals, similar to other findings in the literature. Although data are sparse, elevated aRRs for auditory and menstrual outcomes may warrant further analysis.

**Disclosures:**

**Maria Sundaram, PhD, MSPH**, GlaxoSmithKline: Grant/Research Support **Joshua Petrie, PhD**, CSL Seqirus: Grant/Research Support **Gabriele Neumann, Ph.D.**, FluGen: US-20110150925-A1, US-20100021499-A1, US 8,163,523 B2|FluGen: Ownership Interest|FluGen: Stocks/Bonds **Edward Belongia, MD**, Seqirus: Grant/Research Support **Huong McLean, PhD, MPH**, Seqirus: Grant/Research Support

